# Harvest and natural predation shape selection for behavioural predictability in male wild turkeys

**DOI:** 10.1111/1365-2656.70157

**Published:** 2025-10-15

**Authors:** Nick A. Gulotta, Patrick H. Wightman, Bret A. Collier, Michael J. Chamberlain

**Affiliations:** ^1^ Warnell School of Forestry and Natural Resources University of Georgia Athens Georgia USA; ^2^ School of Renewable Natural Resources Louisiana State University Baton Rouge Louisiana USA

**Keywords:** behavioural predictability, behavioural syndrome, behavioural type, harvest‐induced selection, natural selection, residual intra‐individual variation

## Abstract

1. Previous research has shown that traits increasing prey detectability, such as using open areas, higher activity levels and using areas frequented by hunters, reduce survival rates and are considered risk‐taking behaviours. Despite extensive studies on how harvest‐induced and natural selection impact average phenotypic expression, the influence of these factors on behaviour predictability remains unclear.
2. Using a double hierarchical generalized linear modelling framework, we assessed variation in both average behavioural expression and predictability in male wild turkeys (*n* = 108) and explored how it related to susceptibility to harvest and predation.
3. Our analysis revealed individual variation in predictability linked to risk‐taking and activity (average speed—CVp = 0.28, 95% CrI = 0.23–0.33), with some males exhibiting more consistent behaviour than others. We also identified a behavioural type–predictability syndrome, where riskier individuals were more predictable, and more active individuals were less predictable (Average speed—*r* = 0.71, 95% CrI = 0.59–0.81). Additionally, individuals became more predictable and occurred closer to risky areas during the hunting season, coinciding with peak mortality for male wild turkeys.
4. Our data suggest harvest‐induced and natural selection on both behavioural types and behavioural predictability. Riskier behaviour types were more predictable in their behavioural expression and were more likely to be killed by both hunters and predators. We also found evidence that hunters selectively targeted individuals that were more active and unpredictable in their activity patterns, whereas predators preferentially targeted turkeys exhibiting less active, more predictable behaviours. Our findings suggest that adopting a cautious, sit‐and‐wait tactic may help male wild turkeys detect and avoid hunters, but may increase their vulnerability to predators that use a combination of visual and olfactory cues, along with area‐restricted search behaviours, to locate prey.
5. Our findings highlight the potential for harvest‐induced selection to influence the behavioural composition of male wild turkeys, which has implications for management and conservation strategies.

## Graphical abstract

Male wild turkeys with riskier behavioural types were more predictable in their behavioural expression and more likely to be killed by both hunters and predators. If risk‐taking behaviour is heritable, selective removal of riskier and more predictable individuals may reduce harvest success over time, making wild turkeys increasingly difficult for hunters to detect and harvest.

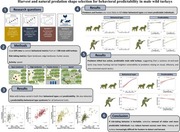

## 1 INTRODUCTION

Contemporary literature demonstrates a growing recognition that individuals differ in their average behavioural expression (i.e. behavioural types), and such differences have been linked to individual fitness (Dingemanse & Réale, 2005; Wolf & Weissing, 2010). Predators play an important role in shaping individual fitness by exerting selective pressures on prey phenotypes, with research consistently showing predators selectively target certain behavioural types that are easier to exploit (Lapiedra et al., 2018). Similarly, numerous studies within the fisheries literature (Andersen et al., 2018; Biro & Post, 2008; Monk et al., 2021; Olsen et al., 2012) and in terrestrial systems (Madden & Whiteside, 2014; Ofstad et al., 2020) have revealed that humans exert selective pressures on behavioural types through a process known as harvest‐induced selection. Studies on harvest‐induced selection have a rich history in the fisheries literature, with two notable studies that examined the influence of harvest‐induced selection and natural selection simultaneously (Edeline et al., 2007; Monk et al., 2021). Despite a substantial body of research pertaining to harvest‐induced selection on behavioural types, we still lack understanding about whether harvest‐induced selection and natural selection impact other hierarchical levels of behavioural variation that are tied to individual differences in behavioural consistency.

Individuals not only differ in their average behavioural expression (i.e. behavioural types) but they also vary in the consistency of their behavioural expression, referred to as behavioural predictability (Biro & Adriaenssens, 2013; Cleasby et al., 2015; O'Dea et al., 2022; Westneat et al., 2015). Predictability was traditionally thought of as a measure of within‐individual error, but recent empirical studies have demonstrated that predictability could represent an additional personality trait since it is both repeatable and heritable (Briffa et al., 2013; Hertel et al., 2021; Martin et al., 2017; O'Dea et al., 2022; Prentice et al., 2020). For instance, movement behaviour studies on barn owls (*Tyot alba*) and brown bears (*Ursus arctos*) have demonstrated a continuum of predictability among individuals, with some exhibiting low within‐individual variation and specializing in certain traits (i.e. more predictable), while others show greater within‐individual variation and are more variable in their behavioural expression (i.e. unpredictable) (Cain et al., 2023; Hertel et al., 2021). The degree of predictability within a specific behavioural trait can correlate with an individual's average behavioural expression, creating what is known as a behavioural type–predictability syndrome (Cleasby et al., 2015; Hertel et al., 2020; O'Dea et al., 2022). For example, research on crocodiles (*Crocodylus porosus*) that assessed variation in monthly site fidelity found that behavioural types with greater site fidelity exhibited lower within‐individual variation and were more predictable in their trait expression (Baker et al., 2024). Furthermore, Cain et al. (2023) found fitness implications of predictability, where individuals more predictable in their movement (i.e. max‐displacement) had smaller home ranges and lower survival than unpredictable individuals. To our knowledge, the Cain et al. (2023) study is the only empirical work that has evaluated survival consequences of predictability in a natural field setting, with the caveat that most mortalities reported in that study were caused by vehicle strikes. As such, we currently lack studies that have examined selection on predictability from natural sources (i.e. predators) and human harvest, which results in a lack of testable predictions and theory. The absence of studies surrounding selection on predictability is surprising, given that within‐individual variation explains a greater proportion of the variation in behaviour compared to the average phenotypic expression (behavioural types) (Bell et al., 2009).

A recent review detailed a framework for the influence of harvest‐induced selection on behavioural traits, highlighting that harvest targets certain behavioural types (Leclerc et al., 2017). For instance, it is predicted that individuals exhibiting risky behavioural types that increase their visibility to hunters, such as frequenting open areas, showing greater activity tendencies and using areas disproportionately frequented by hunters, are likely to experience reduced survival rates. Likewise, numerous studies have revealed that predators commonly target individuals with risk‐taking behavioural types (Dhellemmes et al., 2021; Lapiedra et al., 2018). Specifically, in environments devoid of predators, brown anoles (*Anolis sagrei*) that displayed greater risk‐taking tendencies were characterized by greater use of open areas had increased survival rates, whereas those with similar traits on predator‐rich islands suffered decreased survival (Lapiedra et al., 2018). Consequently, traits that heighten prey detectability reduce individual fitness by promoting selection of high‐risk behavioural types, which are more easily exploited by humans or predators (Ciuti et al., 2012; Madden & Whiteside, 2014; Ofstad et al., 2020). However, several studies have noted that unpredictability might be advantageous for risky behavioural types by limiting the ability of predators to cue in on routine behaviours (Mitchell et al., 2021), leading to correlations that exist between an individual's behavioural type and their degree of predictability (Cain et al., 2023; Hertel et al., 2021). Likewise, it is plausible that similar findings could be extended to harvested species, with individuals that display greater levels of unpredictability being harder to detect and harvest by hunters, but no published studies have attempted to investigate the relationship between predictability and harvest‐induced selection.

We sought to fill these knowledge gaps by assessing harvest‐induced selection and natural selection on the predictability of behaviour in male wild turkeys (*Meleagris gallopavo*). Wild turkeys provide a unique species to evaluate harvest‐induced selection and natural selection since males are harvested during the reproductive period, which is when natural predation is also highest (Wightman et al., 2024). Wild turkeys are an upland game bird that use a polygamous‐promiscuous mating system where females select the most attractive mates for copulation (Dickson, 1992), and males often engage in risk‐taking behaviours to attract and secure mating opportunities. For instance, males often use open landcover for vocalizing and displaying, which increases the chance of attracting receptive females at a greater distance while also facilitating detection by both predators and hunters (Barwick & Speake, 1973; Dickson, 1992; Ey & Fischer, 2010; Godwin et al., 1994; Koda et al., 2008). In fact, prior research has shown that male wild turkeys tend to occur closer to open landcover during the hunting season (Gulotta et al., 2024), a pattern that aligns with hunter behaviour, as hunters frequently employ tactics such as calling and the use of decoys to attract male wild turkeys near open areas. Similarly, female wild turkeys depend on edge landcover for key resources during the reproductive period (Wood et al., 2018; Wood et al., 2019; Yeldell et al., 2017), attracting males in search of food and mating opportunities, but these areas also pose significant risks as they serve as travel corridors for many of the species' primary predators, including hunters (Gerrits et al., 2020; Gross et al., 2015; McNitt et al., 2020; Wightman, Martin, Kohl, Collier, et al., 2023). Additionally, research has shown that while male wild turkeys generally reduce gobbling activity and avoid areas where hunters park their vehicles before entering walking trails (Wightman, Martin, Kohl, Rushton, et al., 2023), individuals with riskier behavioural types tend to remain near these hunter access points despite the associated risks (Gulotta et al., 2024).

While these landcover preferences provide access to important resources such as mates and food, they also expose individuals to varying levels of risk, with previous research showing that male wild turkeys with riskier behavioural types experience lower survival rates from both predators and hunters compared to shyer individuals that avoid high‐risk areas (Gulotta et al., 2024). Additionally, males that exhibit more active behavioural types, as reflected in faster average hourly speeds, tend to have greater survival rates from predators than those that travel at slower speeds (Gulotta et al., 2024). However, despite the perceived dangers of these behaviours, we still lack an understanding as to how the predictability of these traits impacts the survival of male wild turkeys. Our objectives were to (1) assess individual variation in the predictability of risk‐taking and activity, (2) evaluate if the average behavioural expression (behavioural types) is correlated with predictability forming a behavioural type–predictability syndrome, and (3) assess if variation in behavioural types and behavioural predictability predicts survival outcomes from hunters and predators. We hypothesized that both hunters and predators would non‐randomly select behavioural phenotypes that were easier to exploit, and both would target individuals with more predictable behaviour. Based on previous findings that riskier behavioural types experience greater rates of harvest and predation (Gulotta et al., 2024), we predicted that individuals consistently engaging in high‐risk behaviours would exhibit greater behavioural predictability, making them more detectable and vulnerable to hunters and predators. Similarly, we predicted that more active behavioural types, characterized by faster movement and reduced rates of predation (Gulotta et al., 2024), would display greater behavioural unpredictability, making them harder for predators to detect compared to slower moving individuals, who are likely more predictable in their behaviour.

## 2 MATERIALS AND METHODS

### 2.1 Data collection

We captured wild turkeys from January to March of 2014–2023 using rocket nets baited with cracked corn at two study sites, referred to as Georgia and South Carolina (see S1 for more information on study sites). Upon capture, we determined the sex of individuals based on plumage coloration and assessed age, categorizing them into two age classes (Adult, Juvenile) using the barring patterns on the ninth and tenth primary feathers (Dickson, 1992). Each individual was then fitted with a remotely downloadable backpack‐style GPS‐VHF‐UHF transmitter (Biotrack Ltd., Wareham, Dorest, UK and e‐obs GmbH, Gruenwald, Germany). To accommodate GPS transmitter battery constraints, we collected data for approximately 5 months on each wild turkey, focusing on the period from 1 March to 31 July. This timeframe encompassed the entire breeding and hunting seasons, during which male wild turkeys experience greater mortality risks (Wightman et al., 2024). The hunting seasons began in the first 2 weeks of April and ended in the first 2 weeks of May, with no hunting allowed in the fall (see S1 for more details). Hunters could use shotguns or various types of archery equipment like crossbows, compound bows and recurved bows, but the use of rifles or handguns was prohibited. While it was legal to harvest males from both age classes, the hunting regulations prohibited the harvesting of females and typically, hunters targeted adult males (Wightman et al., 2024).

We programmed GPS transmitters to record locations hourly from 05:00 to 20:00, with some instances of 15‐ and 30‐min location intervals for specific study site objectives (see S1 for more information), and one roost location at midnight. We located each bird monthly to download GPS data and monitored survival status three to five times per week. We combined all predator‐related deaths due to insufficient sample sizes and certainty for analysing survival rates specific to predator guild. Additionally, wild turkeys that died without visible trauma were sent to the Southeastern Cooperative Wildlife Disease Study (SCWDS) at the University of Georgia, where necropsies and x‐rays were performed. Data were truncated for birds found dead or harvested by hunters to include only points when the individual was alive, and we cleaned raw GPS data to exclude any points that did not have adequate satellite coverage (dilution of precision values >7; Gupte et al., 2022). All ethics, capture, handling and marking procedures were approved by the Institutional Animal Care and Use Committee at the University of Georgia (#A2019 01‐025‐R2) and Louisiana State University (#A2014‐13, 2015‐07 and 2018‐13).

### 2.2 Behavioural data

Juvenile males were rarely harvested in our study populations, with hunters primarily targeting adults (Wightman et al., 2024). Thus, we analysed GPS data from adult male wild turkeys, focusing on three risk‐taking metrics (distance to edge landcover, distance to open landcover, distance to hunter access) and one activity metric (average hourly speed). Using United States Department of Agriculture (USDA) National Agricultural Statistical Service 30 × 30 m raster layers, we mapped forested (pine, hardwood, shrub and mix pine/hardwoods) and open landcover types, incorporating road data from Georgia Department of Natural Resources, South Carolina Department of Natural Resources and United States Geological Survey to characterize primary and secondary roads (see S2 for more information on characterization of road types). The USDA cropland data layers are updated yearly and can track changes in forest cover resulting from ongoing timber harvest activities, a factor relevant to both study sites. We established an edge landcover feature by identifying areas where forested raster layers bordered open raster layers and both road types. We then used ArcGIS to generate a distance‐based raster, and for the rest of the analysis, we used R version 4.3.1 (R Development Core Team, 2022) along with the raster (Hijmans et al., 2015) and sf (Pebesma, 2018) packages to compute distances from GPS points to these features. A distance‐based approach allowed us to achieve continuous behavioural measurements instead of relying on a classification approach (Conner et al., 2003).

We calculated the nearest distance to hunter access points where the general public could park and reach hunting areas, assuming that areas farther from these points would have less hunting activity (Gerrits et al., 2020; Gross et al., 2015; Wightman, Martin, Kohl, Collier, et al., 2023). For each distance metric (distance to open landcover, distance to edge landcover, distance to hunter access points), we computed the average daily distance for each male wild turkey using these metrics for further analysis. Moreover, to assess activity (i.e. average hourly speed), we computed the total daily distance travelled by each male wild turkey using the adehabitatLT package (Calenge, 2011) and sp package (Pebesma & Bivand, 2005). To obtain the average hourly speed for each individual, we divided the total daily distance by 15 (hours of data collection per day).

### 2.3 Statistical analysis

**FIGURE 1:**
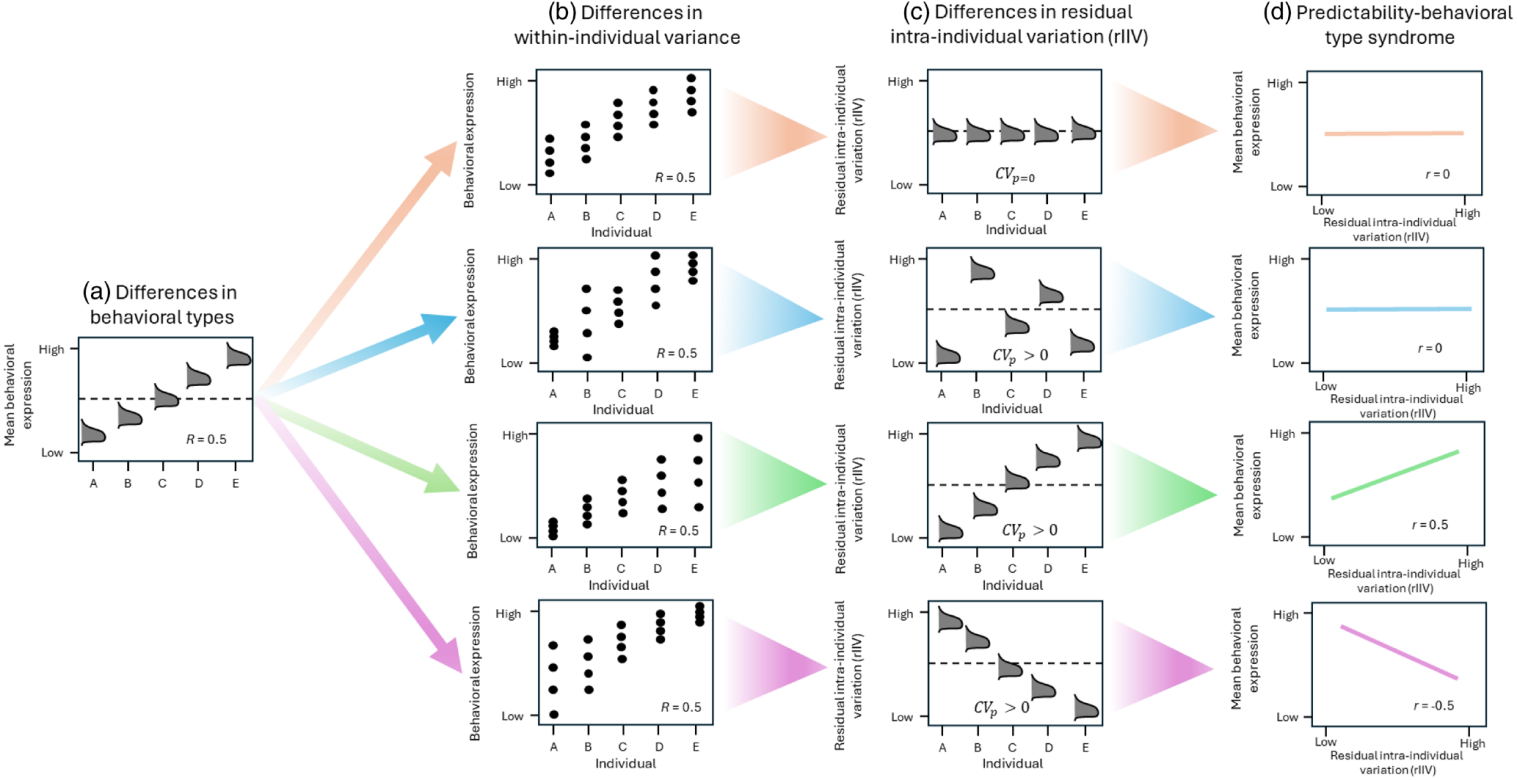
Schematic of hierarchical behavioural variation and possible relationships among an individual's average phenotypic expression (personality) and the variability around the mean behavioural expression (behavioural predictability) adapted from Hertel et al. (2020). (a) Differences in behavioural types—each posterior distribution represents an individual's mean behavioural expression relative to the population mean (dashed line). *R* represents the repeatability of the behavioural trait of interest, or the amount of phenotypic variation explained by among‐individual differences. (b) Differences in within‐individual variance—the continuum of behavioural types and repeatability (*R*) estimates could have the same values across various within‐individual variance structures. Each panel represents a different scenario where *R* = 0.5 but within‐individual variance structure could be equal (orange) or vary among‐individuals (blue, green, purple). (c) Differences in residual intra‐individual variance—predictability‐related differences among individuals is predictability measured as residual intra‐individual variation (rIIV). Individuals can show no among‐individual variation in rIIV (orange; CV_p_ = 0) and not differ from the population level measure of rIIV (dashed line). Conversely, a continuum of unpredictable (high rIIV) and predictable individuals could be observed when the coefficient of variation in predictability (CV_p_) is greater than 0 (blue, green, purple). (d) Behavioural type‐predictability syndrome—variation in behavioural types that could positively covary (*r*) with predictability forming a syndrome (green), negatively covary forming a syndrome (purple) or show no correlation (orange, blue).

To test our objectives surrounding the predictability of behaviour, we constructed four different double hierarchical generalized linear models (DHGLM) that corresponded to each behavioural trait (distance edge landcover, distance open landcover, distance to hunter access points, average hourly speed) using the R package brms (Bürkner, 2017). DHGLMs allow for the simultaneous estimation of both a mean and dispersion model (otherwise known as a residual model), and fixed and random effects can be fit into both models (Cleasby et al., 2015; O'Dea et al., 2022). Therefore, in a singular modelling framework, DHGLMs allow for the estimation of whether individuals differ in the mean behavioural expression (i.e. behavioural types; estimated in the mean model; Figure 1) and whether individuals differ in their residual intra‐individual variation (rIIV) around their behavioural mean (i.e. predictability; estimated by the dispersion model; Figure 1). Furthermore, numerous studies have emphasized that GPS‐based movement data are well suited for the application of DHGLMs, as data on individual movement behaviour can rapidly accumulate into large datasets, providing strong statistical power to detect individual variations in predictability (Hertel et al., 2020; Hertel et al., 2021).

We then categorized the GPS data into three distinct time frames: the pre‐hunt stage (March 1 to the start of the hunting season), the hunt stage (during the hunting season) and the post‐hunt stage (the day after the hunting season ended until July 31). For the mean model, we fit behavioural trait as the response, fixed effects of hunting stage (pre‐hunt, hunt, post‐hunt), study site (Georgia, South Carolina), number of GPS fixes per day and a random intercept for individual ID. We included fixed effects of study site to account for site variation, although our objectives did not involve describing or estimating site‐specific variation. To reduce skew from low GPS fix days and ensure daily distance estimates were based on sufficient data (see Figures S1 and S2), we excluded days with fewer than 10 GPS fixes and removed days when individuals travelled less than 100 m (based on GPS error in each cardinal direction). We also included the number of GPS fixes per day as a fixed effect to account for variability in the number of fixes per day for each individual. For the dispersion models for each behavioural trait, we fitted the same fixed effects and random effects as we did in the mean model. Similar to our mean model, we controlled for site variation in our dispersion model, but site‐specific variation in behavioural predictability was not within the scope of our objectives. In addition, for each behavioural trait, we estimated the correlation between an individual's average behavioural expression (i.e. behavioural type) and predictability, otherwise known as behavioural type–predictability syndrome (Figure 1). By modelling the aforementioned behavioural type–predictability syndrome, we were able to determine whether individuals who were riskier on average were more predictable (low rIIV) or unpredictable (high rIIV) in their behavioural expressions (Figure 1). We also estimated repeatability for each behavioural trait, which describes how much of the phenotypic variation is explained by individual differences in mean behaviour (i.e. behavioural types). We also estimated individual variation in predictability by calculating the coefficient of predictability (CV_p_), which is a standardized population‐level estimate (Figure 1). Lastly, we extracted the posterior distribution of the BLUP (best linear unbiased predictors) of behavioural types and rIIV values for all individuals for use in survival analysis (see below).

Inferences about residual variation can yield misleading outcomes if data are skewed prior to analysis (Baker et al., 2024; Hertel et al., 2021; Prentice et al., 2020; Tatliyer et al., 2019). Therefore, to ensure normality, we performed a square root transformation on distance to edge landcover, distance to open landcover and distance to hunter access. Average hourly speed was left raw since data were normally distributed. After transforming response variables, we standardized all response variables (mean = 0, SD = 1), and then analysed each one using a Gaussian distribution and the default prior in the R package brms (Bürkner, 2017). For each model, we ran 15,300 iterations with a burn‐in of 300, a thinning interval of 15, and we estimated two Markov chains. To confirm that models converged, we computed Rhat values (Rhat values <1.1 confirm convergence) and visually inspected trace and density plots (Bürkner, 2017). We defined effects as significant if credible intervals (CrI) do not overlap zero, or if the probability of direction (PD) is ≥89% for effects whose CrI overlapped zero (Makowski et al., 2019). Effects that overlapped zero but had a PD < 89% were not considered significant. For all DHGLMs, we report the median of the posterior distribution, 95% CrI and probability of direction to assess significance.

#### 2.3.1 Survival analysis

We applied a generalized linear model (GLM) with a binary survival outcome to assess selection on both behavioural predictability (rIIV) and mean behaviour (behavioural types), using the posterior distribution of BLUP values extracted from DHGLM models (Haave‐Audet et al., 2023; LaRocque et al., 2023; Mathot et al., 2022). By iterating across the entire distribution of rIIV and mean behavioural values, we accounted for uncertainty in behavioural measures and avoided the individual gambit, as highlighted in numerous studies (Hadfield et al., 2010). Incorporating the uncertainty around our behavioural measures allowed us to generate 2000 unique GLMs per behavioural trait, with each model estimating the relationship between either behavioural predictability or mean behaviour and survival outcomes. We then calculated the total effect size from these 2000 estimates, reporting the median effect size and 95% CrI for the relationship between each trait and survival. We interpreted a positive log‐odds ratio as evidence that individuals with greater rIIV (more unpredictable) or greater mean behavioural values had increased survival, whereas a negative log‐odds ratio suggested that greater rIIV or greater mean behavioural values were associated with reduced survival.

To assess if behavioural predictability or mean behaviour was selected against by hunters, we subset our data to include individuals that either survived or were harvested in the year they were monitored (*n* = 64 survived, *n* = 27 harvested). We then fitted survival as a binary response and separately included either the BLUP of rIIV or the BLUP of mean behaviour as a fixed effect, applying the same iterative modelling approach as described above. Similarly, we quantified selection imposed by natural sources (predators) by including only individuals that survived or were predated in the year they were monitored (*n* = 64 survived, *n* = 17 predated), again evaluating selection on both behavioural predictability and behavioural types using identical methods. Lastly, to generate pooled selection estimates, we combined both sources of mortality (harvest, predation) and followed the same approach as stated above (*n* = 64 survived, *n* = 44 dead).

## 3 RESULTS

### 3.1 Mean model

**FIGURE 2:**
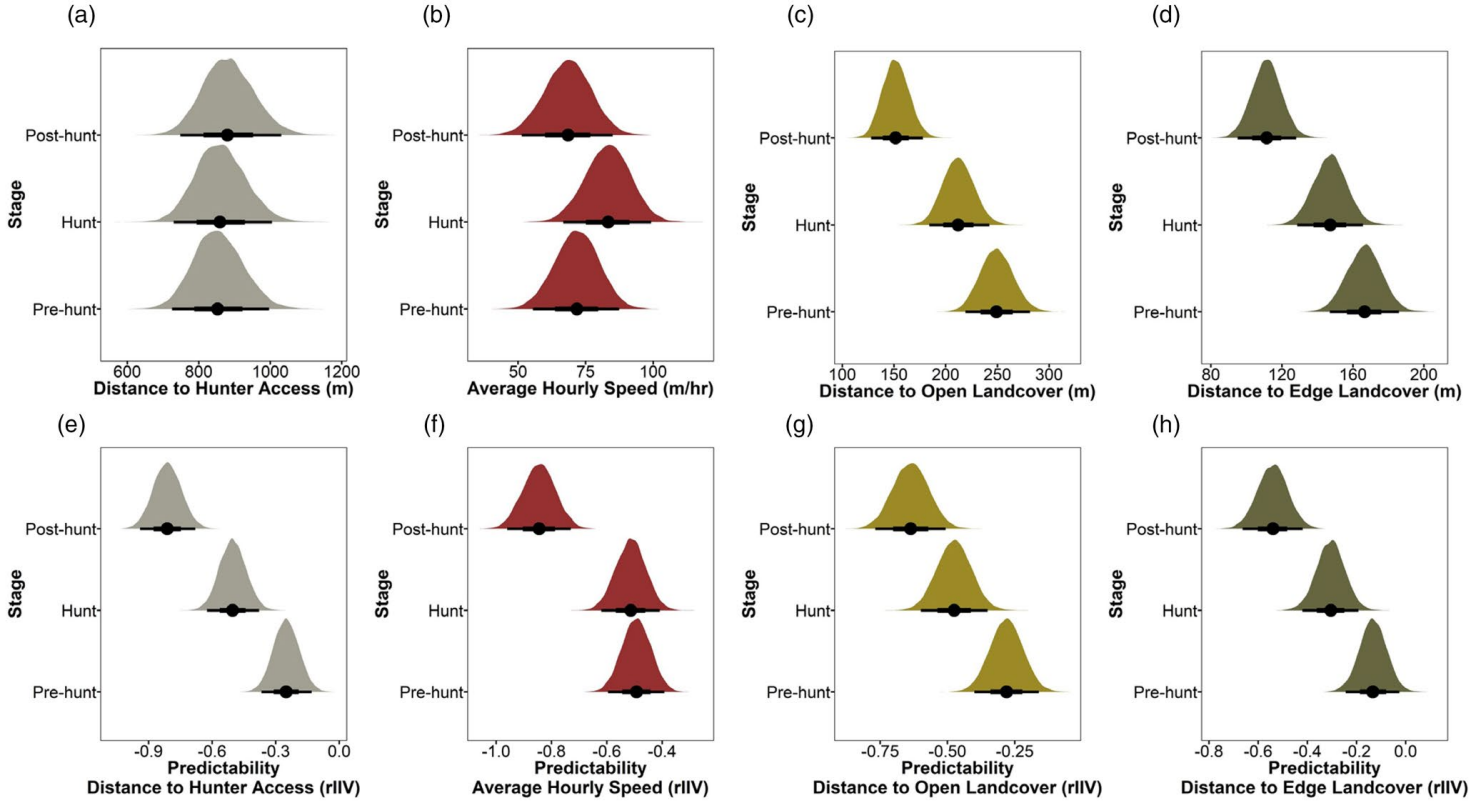
Findings from double hierarchical generalized linear model (DHGLM) assessing the effect of hunting stage for adult male wild turkeys (*n* = 108) during 2014–2023. (a–d) Fixed effect estimates from the mean model. (e–h) Fixed effect estimates from the dispersion model. (a, e) Distance to hunter access, (b, f) average hourly speed, (c, g) distance to open landcover, (d, h) distance to edge landcover. We interpreted lower values of residual intra‐individual variation (rIIV) as more predictable behavioural expression and greater values of rIIV as more unpredictable behavioural expression.

We used data on 108 males and had 9403 daily measures for each behavioural trait of interest (Pre hunt = 3217, Hunt = 2686, Post Hunt = 3500) and an average of 87 repeated measures per individual (minimum = 6, maximum = 155). We found for each behavioural trait moderate to high repeatability, which confirmed the existence of consistent among‐individual differences in risk‐taking and activity (distance to hunter access—*r* = 0.51, 95% CrI = 0.42, 0.60; average speed—*r* = 0.45, 95% CrI = 0.36, 0.53; distance to open landcover—*r* = 0.43, 95% CrI = 0.34, 0.52; distance to edge landcover—*r* = 0.31, 95% CrI = 0.24, 0.39). We also found that individuals occurred closer to risky areas, such as distance to open and edge landcover, during the hunting season (Table 1; Figure 2). Conversely, for another measure of risk‐taking (distance to hunter access), we found no differences among the stages (Table 1; Figure 2). Lastly, we found that individuals were more active during the hunting season compared to the pre‐hunt stage (Table 1; Figure 2).

**TABLE 1:** Results from double hierarchical generalized linear model (DHGLM) of sources of variation in risk‐taking behaviours and exploration for adult male wild turkeys (*n* = 108).

	Distance to hunter access		Average speed		Distance to open landcover		Distance to edge landcover	
**MEAN MODEL**								
**Fixed effects**	***β* (95% CI)**	**PD (%)**	***β* (95% CI)**	**PD (%)**	***β* (95% CI)**	**PD (%)**	***β* (95% CI)**	**PD (%)**
Intercept	0.23 (0.03, 0.44)	98.62	−1.37 (−1.54, −1.21)	100	0.07 (−0.11, 0.25)	77.29	0.57 (0.40, 0.73)	100
Stage								
Hunt	0.01 (−0.02, 0.04)	75.46	0.12 (0.08, 0.16)	100	−0.22 (−0.26, −0.19)	100	−0.17 (−0.20, −0.14)	100
Post‐hunt	0.03 (0.00, 0.06)	98.77	−0.16 (−0.20, −0.11)	100	−0.41 (−0.45, −0.37)	100	−0.34 (−0.37, −0.31)	100
Site	−0.61 (−0.91, −0.31)	100	0.12 (−0.10, 0.35)	86.52	0.48 (0.22, 0.74)	99.99	−0.87 (−1.10, −0.64)	100
Fixes	0.00 (0.00, 0.00)	64.34	0.05 (0.04, 0.05)	100	−0.00 (−0.01, 0.00)	99.80	0.00 (0.00, 0.00)	60.97
**Random effects**	***σ* (95% CrI)**		***σ* (95% CrI)**		***σ* (95% CrI)**		***σ* (95% CrI)**	
Individual ID	0.65 (0.48, 0.83)		0.30 (0.22, 0.39)		0.44 (0.32, 0.57)		0.35 (0.26, 0.45)	
Residual	0.61 (0.47, 0.76)		0.37 (0.30, 0.45)		0.57 (0.44, 0.72)		0.77 (0.60, 0.93)	
**Repeatability**	***r* (95% CrI)**		***r* (95% CrI)**		***r* (95% CrI)**		***r* (95% CrI)**	
Individual ID	0.51 (0.42, 0.60)		0.45 (0.36, 0.53)		0.43 (0.34, 0.52)		0.31 (0.24, 0.39)	
**DISPERSION MODEL**								
**Fixed effects**	***β* (95% CI)**	**PD (%)**	***β* (95% CI)**	**PD (%)**	***β* (95% CI)**	**PD (%)**	***β* (95% CI)**	**PD (%)**
Intercept	−0.25 (−0.37, −0.13)	100	−0.49 (−0.60, −0.39)	100	−0.28 (−0.40, −0.16)	100	−0.13 (−0.24, −0.03)	99.17
Stage								
Hunt	−0.25 (−0.30, −0.21)	91.64	−0.02 (−0.06, 0.02)	83.12	−0.19 (−0.24, −0.15)	100	−0.17 (−0.22, −0.13)	100
Post‐hunt	−0.31 (−0.35, −0.26)	100	−0.33 (−0.37, −0.29)	100	−0.16 (−0.21, −0.12)	100	−0.24 (−0.28, −0.19)	100
Site	−0.09 (−0.22, 0.04)	91.64	0.06 (−0.05, 0.18)	85.89	0.05 (−0.09, 0.18)	75.25	−0.46 (−0.58, −0.34)	100
Fixes	−0.01 (−0.01, −0.01)	100	0.01 (0.01, 0.01)	100	−0.01 (−0.01, 0.00)	100	0.00 (−0.01, 0.00)	99.09
**Random effects**	***σ* (95% CrI)**		***σ* (95% CrI)**		***σ* (95% CrI)**		***σ* (95% CrI)**	
_ω_ Individual ID	0.33 (0.28, 0.39)		0.28 (0.23, 0.32)		0.34 (0.30, 0.40)		0.30 (0.26, 0.35)	
**Coefficient of variation in predictability**	**CV**_**p**_ **(95% CrI)**		**CV**_**p**_ **(95% CrI)**		**CV**_**p**_ **(95% CrI)**		**CV**_**p**_ **(95% CrI)**	
Individual ID	0.35 (0.30, 0.41)		0.28 (0.23, 0.33)		0.35 (0.30, 0.41)		0.31 (0.26, 0.36)	
**Correlation between behavioural type‐predictability**	***r* (95% CrI)**		***r* (95% CrI)**		***r* (95% CrI)**		***r* (95% CrI)**	
*r*_intercept individual ID−ω individual ID_	0.25 (0.06, 0.43)		0.71 (0.59, 0.81)		0.59 (0.44, 0.72)		0.67 (0.54, 0.77)	

*Note*: The study period covered 2014–2023. We present results for the repeatability (*r*), coefficient of variation (CV_p_) in predictability and correlations between behavioural types and predictability (*r*_intercept individual ID−ω individual ID_) for each behavioural trait. Repeatability quantifies the amount of variance attributed to among‐individual differences in behaviour, whereas CV_p_ is a standardized population‐level measure of variation in predictability among individuals. We report beta estimates (*β*) as posterior medians, 95% credible intervals and probability of direction (PD). Any estimate that overlapped zero but had a PD ≥89% was interpreted as a significant effect.

### 3.2 Dispersion model

**FIGURE 3:**
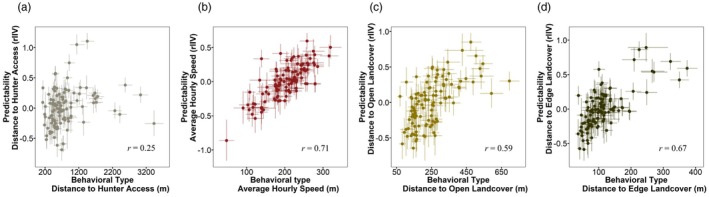
Findings from double hierarchical generalized linear model (DHGLM) assessing the covariation between predictability and behavioural type (individuals' average phenotypic expression) for adult male wild turkeys (*n* = 108) during 2014–2023. Best linear unbiased predictor (BLUP) values for behavioural types and BLUPs for predictability are plotted with 95% CrI (crosshairs), and among‐individual correlations (*r*) between predictability and behavioural type are presented. We interpreted lower distance values for risk‐taking measures (a, c, d) as more risky behavioural types. Greater levels of residual intra‐individual variation (rIIV) were interpreted as more unpredictable individuals, whereas lower levels of rIIV were interpreted as predictable individuals. (a) Distance to hunter access; (c) distance to open land cover; (d) distance to edge landcover—individuals that had more risky behavioural types were more predictable in their behavioural expression, whereas shy individuals had more unpredictable behavioural expressions. (b) Average hourly speed—individuals that travelled at faster speeds on average were more unpredictable, while individuals that travelled slower on average were more predictable.

We detected considerable individual variation in predictability for all behavioural traits (distance to hunter access—CV_p_ = 0.35, 95% CrI = 0.30, 0.41; average speed—CV_p_ = 0.28, 95% CrI = 0.23, 0.33; distance to open landcover—CV_p_ = 0.35, 95% CrI = 0.30, 0.41; distance to edge landcover—CV_p_ = 0.31, 95% CrI = 0.26, 0.36). Likewise, we found evidence for behavioural type–predictability syndromes across all behavioural traits (Table 1; Figure 3). Specifically, for two risk‐taking measures, we found strong positive correlations, indicating that riskier individuals were more predictable in their behavioural expression, whereas risk‐averse individuals were unpredictable (distance to open landcover—*r*_intercept individual ID−ω individual ID_ = 0.59, 95% CrI = 0.44, 0.72; distance to edge landcover—*r*_intercept individual ID−ω individual ID_ = 0.67, 95% CrI = 0.54, 0.77). We found a weak positive correlation between behavioural types and predictability relative to distance to hunter access (distance to hunter access—*r*_intercept individual ID−ω individual ID_ = 0.25, 95% CrI = 0.06, 0.43). We also found a strong positive correlation between behavioural type and predictability for activity (Average hourly speed—*r*_intercept individual ID−ω individual ID_ = 0.71, 95% CrI = 0.59, 0.81), which we interpreted as more active individuals being more unpredictable in their behavioural expression, whereas individuals that were less active were more predictable in their behavioural expression. For all risk‐taking measures, we observed a trend where individuals were more predictable during the hunting season compared to the pre‐hunt stage (Table 1; Figure 2). However, for activity, predictability did not differ between the pre‐hunt and hunting stages (Table 1; Figure 2).

### 3.3 Survival

**FIGURE 4:**
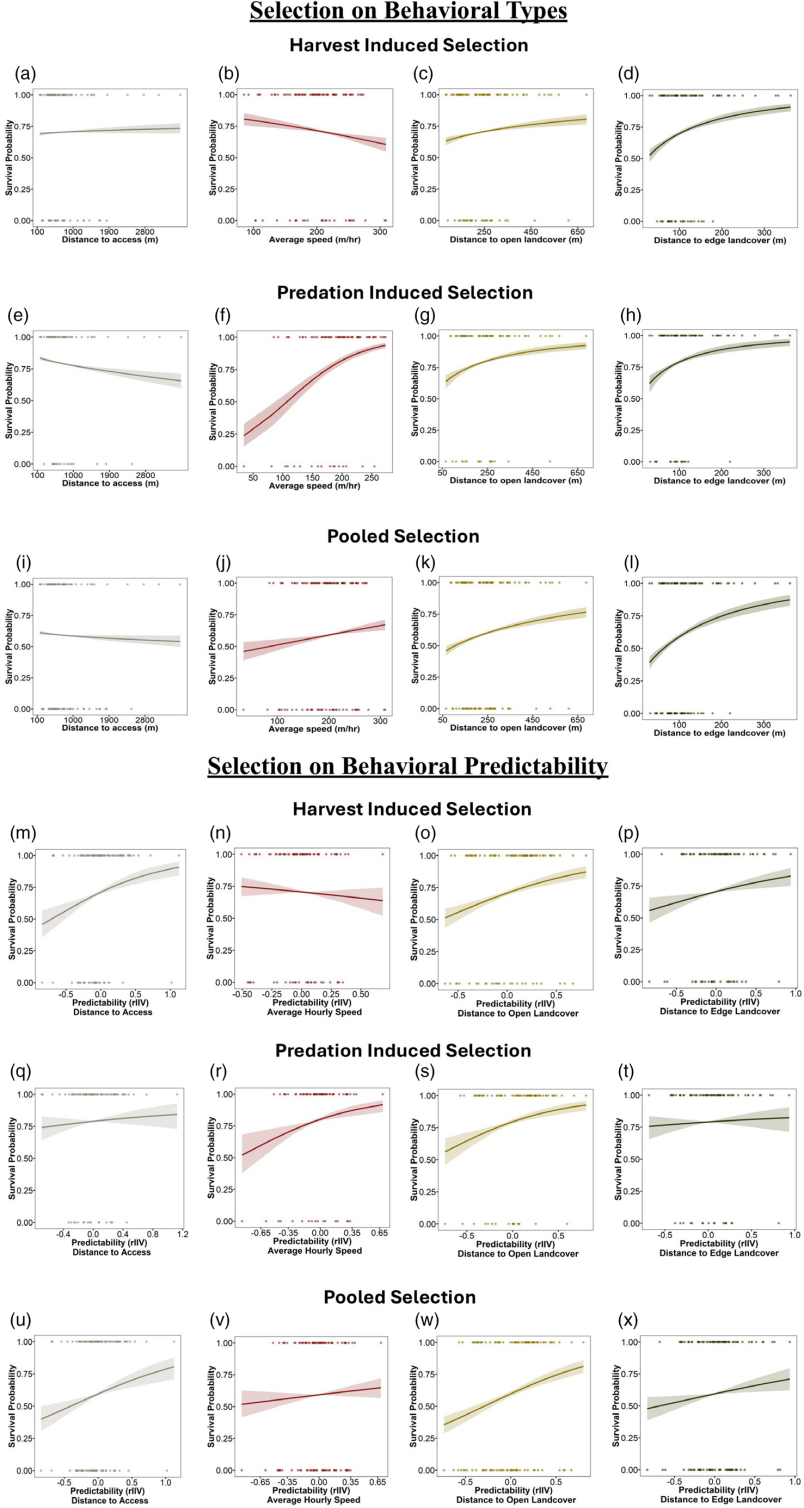
(a–l) Findings from the survival analysis on behavioural types (i.e. mean behavioural expression) of adult male wild turkeys during 2014–2023. (m–x) Findings from the survival analysis on behavioural predictability of adult male wild turkeys during 2014–2023. Each dot represents an individual's mean best linear unbiased predictor (BLUP) value extracted from double hierarchical generalized linear models (a–d, m–p, *n* = 91 individuals; e–h, q–t, *n* = 81 individuals; i–l, u–x, *n* = 108 individuals) and binary survival outcome (1—survived, 0—dead). The posterior median (solid line) and 95% CrI (shaded ribbon) are plotted for the predicted probability of survival. (a–d, m–p) Results of harvest induced selection on behavioural types and behavioural predictability. (e–h, q–t) Results of predator induced selection, and (i–l, u–x) results of pooled survival analysis.

Our findings suggest that harvest‐induced selection acts on both behavioural types and behavioural predictability (Table 2; Figure 4). Across all risk‐taking measures, individuals with more predictable behaviour had lower survival (distance to hunter access—log odds ratio = 1.27, 95% CrI = 0.71, 1.81, PD = 100%; distance to open landcover—log odds ratio = 1.31, 95% CrI = 0.84, 1.81, PD = 100%; distance to edge landcover—log odds ratio = 0.77, 95% CrI = 0.25, 1.27, PD = 99.80%). Likewise, individuals displaying riskier behavioural types, characterized by closer proximity to high‐risk areas, also had reduced survival (distance to hunter access—log odds ratio = 0.05, 95% CrI = −0.02, 0.13, PD = 91.40%; distance to open landcover—log odds ratio = 0.29, 95% CrI = 0.16, 0.41, PD = 100%; distance to edge landcover—log odds ratio = 0.74, 95% CrI = 0.57, 0.93, PD = 100%). Lastly, for activity (average speed), we found evidence that individuals with more active behavioural types (average speed—log odds ratio = −0.43, 95% CrI = −0.67, −0.21, PD = 100%), and individuals that were unpredictable (average speed—log odds ratio = −0.43, 95% CrI = −1.15, 0.29, PD = 89.00%) were more likely to be harvested (Table 2; Figure 4).

**TABLE 2:** Results of survival analysis for each behaviour and mortality source for adult male wild turkeys during 2014–2023.

Fixed effect	Mortality source	Distance to hunter access		Average speed		Distance to open landcover		Distance to edge landcover	
		*β* (95% CrI)	PD (%)	*β* (95% CrI)	PD (%)	*β* (95% CrI)	PD (%)	*β* (95% CrI)	PD (%)
BLUP of behavioural types	Human	0.05 (−0.02, 0.13)	91.40	−0.43 (−0.67, −0.21)	100	0.29 (0.16, 0.41)	100	0.74 (0.57, 0.93)	100
	Predator	−0.23 (−0.31, −0.14)	100	1.56 (1.27, 1.90)	100	0.59 (0.41, 0.79)	100	0.84 (0.56, 1.17)	100
	Pooled	−0.07 (−0.12, 0.00)	98.00	0.30 (0.14, 0.47)	100	0.41 (0.30, 0.52)	100	0.81 (0.63, 1.00)	100
BLUP of rIIV	Human	1.27 (0.71, 1.81)	100	−0.43 (−1.15, 0.29)	89.00	1.31 (0.84, 1.81)	100	0.77 (0.25, 1.27)	99.80
	Predator	0.36 (−0.31, 1.06)	85.30	1.53 (0.66, 2.29)	100	1.51 (0.87, 2.14)	100	0.27 (−0.44, 1.00)	78.40
	Pooled	0.94 (0.46, 1.43)	100	0.37 (−0.16, 0.83)	89.00	1.35 (0.95, 1.75)	100	0.56 (0.13, 1.02)	99.80

*Note*: Survival (1—survived, 0—dead) was fit as the response and best linear unbiased predictor (BLUP) values of behavioural types and residual intra‐individual variance (rIIV) for each individual were fit as a fixed effect using data on adult males (*n* = 108, *n* = 64 survived, *n* = 27 harvested, *n* = 17 predated). Beta estimates (*β*) from the analysis are presented as log‐odds ratios with 95% credible intervals (CrI). We report posterior medians, 95% credible intervals and probability of direction (PD). Any estimate that overlapped zero but had a PD ≥89% was interpreted as a significant effect.

We also found that natural selection shapes both behavioural types and behavioural predictability, although the patterns did not always mirror those of harvest‐induced selection (Table 2; Figure 4). Specifically, individuals exhibiting riskier behavioural types that were closer to open landcover had lower survival (distance to open landcover—log odds ratio = 0.59, 95% CrI = 0.41, 0.79, PD = 100%), as did those who were more predictable in their distance to open landcover (distance to open landcover—log odds ratio = 1.51, 95% CrI = 0.87, 2.14, PD = 100%). Similarly, for distance to edge landcover, riskier behavioural types had lower survival (distance to edge landcover—log odds ratio = 0.84, 95% CrI = 0.56, 1.17, PD = 100%), and while there was a tendency for more predictable individuals to also have lower survival, this effect was not statistically significant (Table 2; Figure 4). In contrast, individuals with riskier behavioural types that occurred closer to hunter access had greater survival (distance to hunter access—log odds ratio = −0.23, 95% CrI = −0.31, −0.14, PD = 100%), but there was no evidence that predictability in distance to hunter access affected survival (Table 2; Figure 4). For activity, faster behavioural types (average hourly speed—log odds ratio = 1.56, 95% CrI = 1.27, 1.90, PD = 100%) and more unpredictable individuals had higher survival (average hourly speed—log odds ratio = 1.53, 95% CrI = 0.66, 2.29, PD = 100%) compared to slower and more predictable individuals. Finally, our pooled analysis revealed a consistent trend across all behavioural traits, where more predictable individuals had lower survival but unpredictable individuals had greater survival (Table 2; Figure 4).

## 4 DISCUSSION

Numerous studies have reported that individuals vary in their perception of risk, forming a continuum from bold to risk‐averse individuals (Dingemanse & Réale, 2005; Wolf & Weissing, 2010), which subsequently influences how individuals interact with risky areas within their home range that are linked to greater mortality risks, such as open areas or proximity to common hunter and predator travel routes (Ciuti et al., 2012; Leclerc et al., 2017; Ofstad et al., 2020). Studies on behavioural predictability suggest that individuals with more predictable and routine‐like behaviour may face a greater risk of predation (Briffa, 2013; Jolles et al., 2019; Stamps et al., 2012), as predators can recognize and exploit predictable movement patterns, but whether hunters similarly exploit more predictable individuals remains unknown. Here, we provide evidence that riskier individuals tend to be more predictable and that, overall, individuals occur closer to risky areas and become more predictable during the hunting season, when male wild turkey mortality is at its peak (Wightman et al., 2024). We also demonstrate that riskier behavioural types and more predictable individuals exhibit lower survival rates from both human harvest and natural selection. Although both behavioural type and predictability were strongly associated with survival, their correlation complicates interpretation, as a behavioural type–predictability syndrome suggests that selection acting directly on one source of variation could indirectly influence the other, making it difficult to identify the primary target of selection. Clarifying the primary target of selection will require creative study designs capable of isolating each form of behavioural variation, such as controlled experiments that manipulate behavioural type or predictability independently to assess their direct effects on fitness. Manipulating predation risk may offer one approach, as varying levels of predation risk have been shown to both induce and disrupt behavioural syndromes (correlation between the mean phenotypic expression of two different behavioural traits) and could elicit a similar effect on behavioural type–predictability syndromes (Dhellemmes et al., 2021).

Prior research has shown that riskier individuals exhibit more rigid behavioural patterns (Bucklaew & Dochtermann, 2021), and our findings further support emerging evidence that riskier individuals also tend to be more predictable (Briffa et al., 2013; Jolles et al., 2019; Prentice et al., 2020), whereas risk‐averse individuals display greater behavioural flexibility. In our study, riskier individuals that stayed closer to open or edge landcover and exhibited more predictable movement patterns had lower survival, as they were more likely to be harvested or killed by predators. Wild turkeys often rely on open and edge landcover for foraging and courtship (Barwick & Speake, 1973; Godwin et al., 1994; Wightman, Martin, Kohl, Collier, et al., 2023; Williams, 1981; Wood et al., 2018), which likely makes them more detectable to both predators and hunters that also use these landcover types. Our other risk‐taking measure, distance to hunter access, which corresponds to areas where hunters park their vehicles and is limited to primary roads across both study sites, exhibited variable selection patterns compared to our other two measures of risk‐taking. While individuals did not alter their distance to hunter access across different hunting stages, they became more predictable in their movements during the hunting season. Our findings indicate that individuals occurring closer to hunter access points were more likely to be harvested. However, despite being statistically significant, we note that the effect size is likely not biologically meaningful (mean distance for harvested individuals = 774 ± 465 m, mean distance for surviving individuals = 817 ± 667 m; Table 2). Regardless, research on other harvested species, including moose and elk, has shown that individuals exhibiting riskier behaviours, such as using open areas and hunter travel routes, face a higher likelihood of harvest (Ciuti et al., 2012; Leclerc et al., 2019; Ofstad et al., 2020). Our study adds to the growing body of evidence that frequenting areas commonly used by hunters increases detectability and mortality risk for game species (Leclerc et al., 2017).

Studies on harvested species, such as deer and elk, have shown that individuals often decrease their activity during the hunting season to minimize encounters with hunters (Ciuti et al., 2012; Little et al., 2016). In contrast, our findings suggest that male wild turkeys increase their activity during the hunting season. We propose that an increase in the average activity level during the hunting season likely functions as a tactic to increase encounter rates with reproductively active females dispersed across the landscape or as a mechanism to mitigate mortality risk. Several authors have suggested that more active individuals face greater harvest risk due to increased detectability (Leclerc et al., 2017; Mysterud, 2011). Our data support such predictions, suggesting that male wild turkeys with predictable, slower movements tend to avoid harvest better than faster, unpredictable individuals. Interestingly, harvest‐induced selection and natural selection exhibited opposing effects in our study. We suggest that opposing selection pressures prompt male wild turkeys to adopt diverse behavioural tactics in response to perceived predation risk. For example, male wild turkeys with predictability slower movements may excel at detecting hunters by sight or sound. Conversely, moving at slower speeds may also correlate with smaller home ranges as shown by Little et al. (2016) on white‐tailed deer (*Odocoileus virginianus*), giving individuals that adopt this tactic better knowledge of hunter activity within their range and enabling them to avoid common travel routes used by hunters. Additionally, a sit‐and‐wait tactic, characterized by slower and predictable movement, can complement the crypsis tactic used by some wild turkeys (Lohr et al., 2020), allowing them to remain motionless and hidden while evading detection by hunters, which could increase their chances of survival. However, our findings indicate that this tactic also exposes individuals to higher predation risk, likely due to predator hunting tactics that rely on olfactory and visual cues, along with area‐restricted‐search techniques (Chamberlain et al., 2021).

Collectively, our findings suggest that harvesting wild turkeys may become increasingly challenging over time if risk‐taking and behavioural predictability are heritable traits (Grzegorczyk et al., 2024; Martin et al., 2017; Prentice et al., 2020). Hunters frequently use edge landcover as a travel corridor to locate wild turkeys, with most hunting activity concentrated within 20–100 m of these areas (Gerrits et al., 2020; Gross et al., 2015), while also using decoys and calls near open landcover to attract males by mimicking reproductively active females. However, if wild turkey populations shift towards individuals that are more unpredictable and avoid high‐risk areas associated with hunters, traditional hunting tactics may become less effective. Furthermore, emerging research has shown that behavioural type–predictability syndromes may be under strong genetic control (Prentice et al., 2020), potentially limiting risk‐prone individuals' ability to modify their behaviour. If other game species exhibit a similar pattern to our findings, where greater risk‐taking and higher predictability are linked to increased mortality from both hunters and predators, populations may gradually become dominated by individuals that are more difficult to detect, characterized by lower risk‐taking and greater behavioural unpredictability. Over time, a shift towards individuals that are more unpredictable and occur farther from areas used by hunters could make harvesting game species more difficult (Leclerc et al., 2017), reducing the effectiveness of traditional hunting tactics.

We also demonstrate that, for both measures of risk‐taking, harvest‐induced selection and natural selection were operating in a similar direction both on the mean phenotypic expression (behavioural type) and the consistency of behavioural expression (predictability). Previous studies have shown that strong selection pressures from harvest‐induced selection and natural selection can lead to directional shifts in trait frequencies over time (Edeline et al., 2007; Kvalnes et al., 2016), a process known as directional selection. We recognize that our work is insufficient to assess directional selection but offer that future efforts should investigate directional selection in the behaviours of highly sought‐after game species, including wild turkeys. We note that most research on directional selection has concentrated solely on physical traits (such as body size, antler and horn size), largely overlooking behavioural traits that could increase an individual's susceptibility to harvest. The lack of research examining directional selection on behavioural traits is surprising, considering that temporal changes in behavioural trait frequencies could complicate harvest, potentially diminishing hunter satisfaction and reducing revenues from hunting licences that support wildlife agencies (Grzegorczyk et al., 2024; Leclerc et al., 2017; Schroeder et al., 2018). Concurrently, temporal shifts in trait frequency could reduce the resilience of game populations to disease and environmental changes, thus adversely affecting their long‐term persistence (Edeline et al., 2007; Grzegorczyk et al., 2024; Kvalnes et al., 2016; Leclerc et al., 2017). We recommend future studies investigate the heritability of behavioural consistency in movement traits, as prior studies have found the predictability of commonly assayed behaviours in field settings to be heritable (Martin et al., 2017).

## AUTHOR CONTRIBUTIONS

Nick Gulotta, Patrick Wightman, Bret Collier and Michael Chamberlain conceived the ideas and designed methodology; Nick Gulotta and Patrick Wightman collected the data; Nick Gulotta was the lead on analysing the data and Patrick Wightman provided support; Nick Gulotta led the writing of the manuscript. All authors contributed critically to the drafts and gave final approval for publication.

## CONFLICT OF INTEREST STATEMENT

The authors declare no conflicts of interest.

## Supporting information


**Figure S1.** Relationship between number of GPS fixes and total distance travelled (m). Plot shows relationship for raw data before exclusion of data with fewer than 10 GPS fixes per day.
**Figure S2.** Relationship between number of GPS fixes and total distance travelled (m). Plot was subset to include only individuals with 10 or more GPS fixes in a day.

## Data Availability

Data available from the Dryad Digital Repository: https://doi.org/10.5061/dryad.79cnp5j8p (Gulotta et al., 2025).
