# Supplementary material for: Harvest and natural predation shape selection for behavioural predictability in male wild turkeys
Source: J Anim Ecol. 2025 Oct 15;94(12):2627–40. doi: 10.1111/1365-2656.70157 (PMC12673237; doi:10.1111/1365-2656.70157)
Supplement: Supplementary file 1 — Figure S1. Relationship between number of GPS fixes and total distance travelled (m). Plot shows relationship for raw data before exclusion of data with fewer than 10 GPS fixes per day. Figure S2. Relationship between number of GPS fixes and total distance travelled (m). Plot was subset to include only individuals with 10 or more GPS fixes in a day. [file JANE-94-2627-s001.docx]

## **SUPPLEMENTARY MATERIAL**

## **Harvest and natural predation shape selection for behavioral predictability in male wild turkeys**

## Nick A. Gulotta^1^, Patrick H. Wightman^1^, Bret A. Collier^2^, Michael J. Chamberlain^1^

## ¹ Warnell School of Forestry and Natural Resources, University of Georgia, Athens, Georgia, USA

^2^ School of Renewable Natural Resources, Louisiana State University, Baton Rouge, LA 70803

**S1. STUDY SITE INFORMATION**

We conducted research on two wildlife management areas (WMAs) and surrounding private lands in the Piedmont region of Georgia, USA. Cedar Creek WMA (CCWMA) was approximately 16,187 hectares, and B.F. Grant WMA (BFGWMA) was about 4,613 hectares, both managed in cooperation with the Georgia Department of Natural Resources - Wildlife Resources Division (GADNR). CCWMA was owned by the United States Forest Service, whereas BFGWMA was owned by the Warnell School of Forestry and Natural Resources at the University of Georgia. We captured birds from 2017-2023 on CCWMA, spring hunting seasons for males generally started the last week of March or first week of April and concluded on 15 May, and hunting was open to the public at the beginning of each hunting season. Landcover on BFGWMA included agricultural fields for hay cultivation and livestock grazing, planted loblolly pine (*Pinus taeda*) stands, mixed pine and hardwood forests, and hardwood forests. BFGWMA's hunting seasons were divided into three phases: youth hunt (approximately March 25 - April 2), 80-person quota hunt (approximately April 3 - April 9), and general public hunting season (approximately April 10 - May 15). For more detailed information on climate and management practices, see Wakefield et al. (2020). To support site specific research objectives, we collected GPS location data from wild turkeys during 2017-2021 using GPS transmitters that recorded hourly locations. During the 2022-2023 field seasons, we deployed e-obs (e-obs GmbH, Gruenwald, Germany) activity-informed GPS-ACC transmitters, which took hourly GPS points regardless of activity, and 15-minute locations when birds were deemed active, based on tri-axial accelerometer data recorded on board the units.

Additionally, from 2014-2018, we conducted work on the Webb WMA complex in Garnett, South Carolina, USA, near the Savannah River. The Webb WMA complex, managed by the South Carolina Department of Natural Resources (SCDNR), encompassed approximately 10,483 hectares and included Hamilton Ridge WMA, Palachacola WMA, and Webb WMA. This area featured a variety of landcover types such as bottomland and upland hardwoods, planted pines (loblolly and longleaf pine—Pinus palustris), mixed pine-hardwood forests, wetlands, and wildlife openings. For further information on climate and management practices, see Chamberlain et al. (2018). The hunting season in South Carolina varied over the five years but generally opened around April 1 and ended at the end of April or the first week of May, with hunting allowed Monday through Saturday but not on Sundays. To support study site–specific objectives, we collected GPS locations from marked wild turkeys at 30-minute intervals throughout the duration of the study.

**S2. ROAD DATA**

We obtained road data from GADNR and SCDNR for roads inside the WMAs and used USGS Tiger/Line data (Topologically Integrated Geographic Encoding and Referencing) to delineate roads outside the WMAs that traversed private lands. We characterized secondary roads as either logging roads and/or unpaved gravel roads that did not allow vehicular access, whereas primary roads were paved/graveled and vehicle access was not limited.

**S3. RELATIONSHIP BETWEEN GPS FIXES AND TOTAL DAILY DISTANCE TRAVELED**

To account for potential biases due to variation in GPS fix rates across days, we evaluated the relationship between the number of fixes and daily distance traveled, which was found to be moderate to low (Figure S1). To minimize skew from days with low fix counts and ensure reliable estimates of daily distance traveled, we retained only days with ≥10 fixes per individual and excluded days when individuals traveled <100 m (Figure S2). All models were run with a fixed effect for the number of daily fixes to account for variation in fix rates among individuals (see ‘Methods’ section in main text).

**FIGURE S1. RAW RELATIONSHIP BETWEEN GPS FIXES AND TOTAL DAILY DISTANCE TRAVELED**


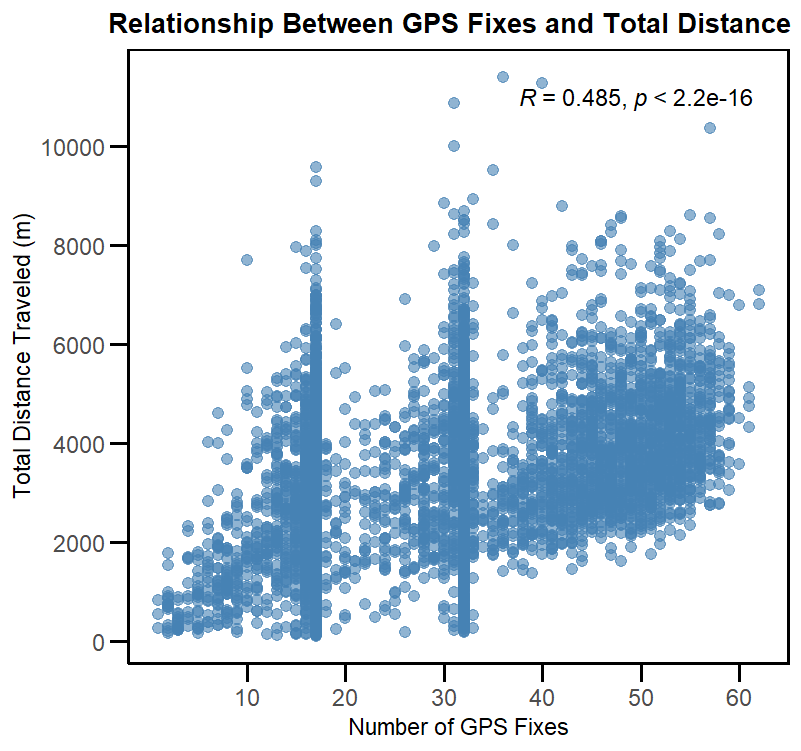


**Figure S1.** Relationship between number of GPS fixes and total distance traveled (m). Plot shows relationship for raw data before exclusion of data with fewer than 10 GPS fixes per day.

**FIGURE S2. RELATIONSHIP BETWEEN GPS FIXES AND TOTAL DAILY DISTANCE TRAVELED SUBSET TO ≥ 10 FIXES IN A DAY.**


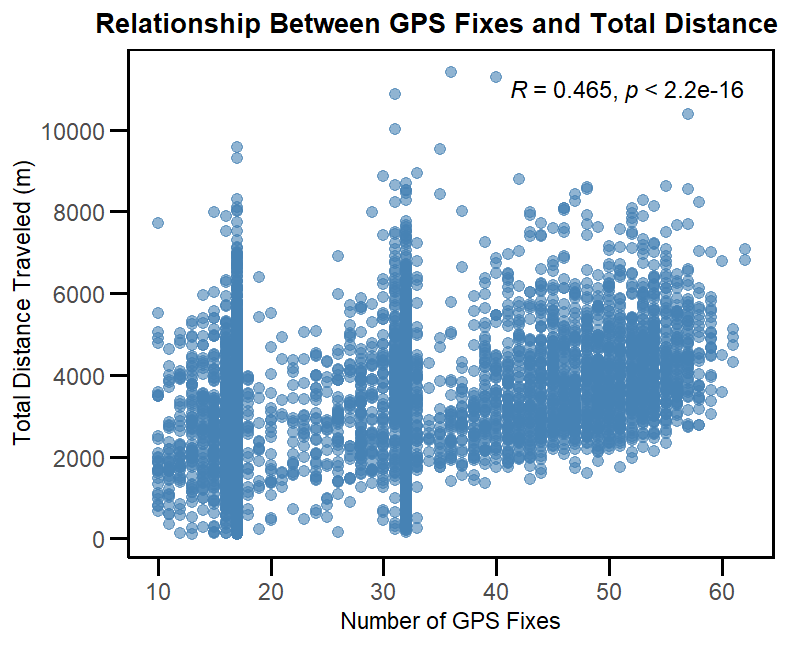


**Figure S2.** Relationship between number of GPS fixes and total distance traveled (m). Plot was subset to include only individuals with 10 or more GPS fixes in a day.

**REFERENCES**

Chamberlain, Michael J, Patrick H Wightman, Bradley S Cohen, and Bret A Collier. 2018. 'Gobbling activity of eastern wild turkeys relative to male movements and female nesting phenology in South Carolina', *Wildlife Society Bulletin*, 42: 632-42.

Wakefield, Calvin T, James A Martin, Patrick H Wightman, Bobby T Bond, D Kevin Lowrey, Bradley S Cohen, Bret A Collier, and Michael J Chamberlain. 2020. 'Hunting activity effects on roost selection by male wild turkeys', *Journal of Wildlife Management*, 84: 458-67.
